# Dissimilarity of individual microsatellite profiles under different mutation models: Empirical approach

**DOI:** 10.1002/ece3.5032

**Published:** 2019-03-19

**Authors:** Evsey Kosman, Jukka Jokela

**Affiliations:** ^1^ Institute for Cereal Crops Improvement Tel Aviv University Tel Aviv Israel; ^2^ ETH Zurich, Department of Environmental Systems Science Institute of Integrative Biology (IBZ) Zurich Switzerland; ^3^ EAWAG Aquatic Ecology Dübendorf Switzerland

**Keywords:** Bruvo's distance, genetic dissimilarity of individuals, infinite alleles model, population structure, SSR markers, stepwise mutation model

## Abstract

Microsatellites (simple sequence repeats, SSRs) still remain popular molecular markers for studying neutral genetic variation. Two alternative models outline how new microsatellite alleles evolve. Infinite alleles model (IAM) assumes that all possible alleles are equally likely to result from a mutation, while stepwise mutation model (SMM) describes microsatellite evolution as stepwise adding or subtracting single repeat units. Genetic relationships between individuals can be analyzed in higher precision when assuming the SMM scenario with allele size differences as a proxy of genetic distance. If population structure is not predetermined in advance, an empirical data analysis usually includes (a) estimating proximity between individual SSR profiles with a selected dissimilarity measure and (b) determining putative genetic structure of a given set of individuals using methods of clustering and/or ordination for the obtained dissimilarity matrix. We developed new dissimilarity indices between SSR profiles of haploid, diploid, or polyploid organisms assuming different mutation models and compared the performance of these indices for determining genetic structure with population data and with simulations. More specifically, we compared SMM with a constant or variable mutation rate at different SSR loci to IAM using data from natural populations of a freshwater bryozoan *Cristatella mucedo* (diploid), wheat leaf rust *Puccinia triticina* (dikaryon), and wheat powdery mildew *Blumeria graminis* (monokaryon). We show that inferences about population genetic structure are sensitive to the assumed mutation model. With simulations, we found that Bruvo's distance performs generally poorly, while the new metrics are capturing the differences in the genetic structure of the populations.

## 1 INTRODUCTION

Microsatellites (simple sequence repeats, SSRs, i.e., tandem repeats of a few nucleotides) still remain popular molecular markers for study of neutral genetic variation. The number of tandems may vary significantly among different individuals due to high mutation rates of microsatellites, and this polymorphism makes microsatellites attractive population genetic markers. Microsatellites are also special among molecular markers in that their repeat structure provides information on the relatedness of alleles. These intrinsic properties of microsatellites make them very powerful for population genetic studies and advocate for their use in the future (Allentoft, Heller, Holdaway, & Bunce, 2015; Chen, Lu, Zhu, Tamaki, & Qiu, 2017; Dufresne, Stift, Vergilino, & Mable, 2014; Nybom, Weising, & Rotter, 2014; Putman & Carbone, 2014) despite extensive application of more recent marker‐rich techniques based on next generation sequencing data.

Two alternative approaches outline how new microsatellite alleles evolve. Stepwise mutation model (SMM) and two‐phase model (TPM) describe microsatellite evolution as some combination of a regular stepwise change of adding or subtracting single or a few repeat units, while infinite alleles model (IAM) assumes a random process in which all possible alleles are equally likely to result from a mutation at a given microsatellite locus. Microsatellite evolution according to the simple SMM assumes that each mutation adds or subtracts (with equal probability) a single repeat unit, potentially leading to a new allele (Jarne & Lagoda, 1996). Assuming that this type of a random walk actually reflects the process of evolutionary change in microsatellites, the relative similarity in allele sizes of microsatellites in two individuals should be a function of the genetic distance between them. Therefore, assessment of similarity or dissimilarity between SSR genotypes of individuals might be more powerful if allele sizes were taken into account (SMM or TPM scenario) rather than just comparing numbers of loci at which the individuals have different alleles (IAM scenario).

In this paper, we consider between‐individual measures of genetic dissimilarity rather than among‐population measures of genetic differentiation. The efficiency of the among‐population differentiation measures based on allele identity (IAM) versus allele size (SMM or TPM) has been previously discussed (e.g., Hardy, Charbonnel, Freville, & Heuertz, 2003). Instead, we focus on how distance between SSR alleles can be used to study between‐individual genetic dissimilarity and subdivision of individuals to groups. Importantly, IAM and SMM (or TPM) models differ essentially in what they assume about the distance between SSR alleles. The consequences of these different assumptions for utility of individual dissimilarity measures are largely unknown and the topic of our paper.

Population genetic studies, where no a priori information on ancestry of the sampled individuals is available, usually analyze the data in following steps.

Step 1. Estimate genetic distance among individuals using the selected measure of dissimilarity.
Step 2. Use the genetic distance estimates to determine the putative among‐individual structure using clustering and/or ordination techniques.
Step 3. Evaluate if the revealed subgroups of individuals can be considered as separate populations.

Here, we developed new tools for Step 1 and tested their performance at Step 2 using data from populations of a freshwater bryozoan (diploid) and two fungal pathogens of wheat (dikaryon and monokaryon).

Pairwise dissimilarity among individuals is the root of many inferences about structure and diversity of a given set of data. Therefore, selection of a proper dissimilarity measure is a key issue of most analysis methods (e.g., UPGMA and NJ dendrograms, NMDS ordination). The first methods applying dissimilarity between SSR profiles required conversion of the microsatellite data to presence–absence data (markers were genotyped as dominant instead of codominant markers). Each allele of a particular size was considered as an independent locus, and an individual binary profile included 1 in the corresponding position in the case of presence of that allele or 0 otherwise. The dissimilarity between the obtained binary profiles was then measured with either the simple mismatch, Jaccard, or Dice index. Application of these indices to profiles with codominant markers is not generally valid even assuming IAM scenario (for details see Kosman & Leonard, 2005). In such analysis, only identity of the alleles at the same SSR locus is considered and mutational processes are ignored. Such treatment leads to loss of potentially important information about the extent of similarity between alleles of different sizes.

The next generation of methods used microsatellite allele size for measuring genetic distances between and differentiation among populations (Goldstein, Linares, Cavalli‐Sforza, & Feldman, 1995a, 1995b; Shriver et al., 1995; Slatkin, 1995). These distances were then also applied for comparison among individuals (e.g., Udupa, Robertson, Weigand, Baum, & Kahl, 1999; Otter, Murray, & Holschuh, 2003). However, when applied to a pair of individuals, they do not seem to work properly because some of them do not distinguish between individuals with different SSR genotypes, while others may discriminate between identical multilocus profiles (see “Discussion”).

The most recently published method was suggested by Bruvo, Michiels, D'Souza, and Schulenburg (2004), and since then it has been the most commonly and increasingly used approach for measuring dissimilarity between multilocus microsatellite genotypes. More specifically, the study received 74 citations in the first 9 years after publication (2013–2004), and 93 citations during the last two years (2017–2018). At present, the method is adopted in number of software packages (e.g., GenoDive, POLYSAT and Poppr) and is one of the recommended measures for dissimilarity analyses, especially when there is variation in ploidy in the study species. Bruvo's method has been applied assuming generalized SMM (Slatkin, 2002) or the two‐phase model (TPM) of DiRienzo et al. (1994). Their method relies on assuming a nonlinear dependence of distance between SSR alleles and size difference between those alleles. We will critically discuss this method in detail demonstrating its inappropriateness for most analyses it is used for (see “Discussion”). Further critique toward Bruvo's distance can be found in Meirmans, Liu, and van Tienderen (2018).

It is still rare that analyses of dissimilarities between individuals with codominant SSR data use information about allele sizes. In most cases, such analyses rely on identity of alleles. Our main objective was to develop new indices of dissimilarity for comparison between SSR genotypes of haploid, diploid, and polyploid individuals. Ideally, these indices reflect true genetic differences between individuals. Analysis of genetic differences between individuals gives more accurate information of the population history, connectedness, mating system, and relatedness of individuals in the population. For example, using allele sizes instead of identity makes it easier to evaluate the proportion of migrants (gene flow), assess the ancestral population size and population recovery from bottlenecks. It is common in population genetic studies to use IAM scenario as it is difficult to know exactly what the accurate mutation models are for the used SSR loci. In that sense, using IAM is considered as a robust and conservative application. We believe that applying IAM scenario to analysis of genetic structure is not always conservative, as it may miss important and useful information and can even be misleading in some cases. Here, we study new indices of dissimilarity in two scenarios, assuming a stepwise mutational process with a constant or variable mutation rate among SSR loci. We compared these indices by analyzing data from individuals of a motile bryozoan *Cristatella mucedo* (diploid) living in lakes of Switzerland, isolates of wheat rust *Puccinia triticina *Eriks. (dikaryon) collected from wheat in seven regions of Russia, and wheat powdery mildew *Blumeria graminis *f. sp. *tritici *(monokaryon) originating from wild and domesticated *Triticum *species in Israel. As it is usually not possible to assume that samples from natural populations conform to one specific mutation model, we compared the topology of UPGMA trees derived using the same data, but based on dissimilarity matrices obtained assuming different mutation models. We expected that if genetic structure of the given populations (relationships between individuals) is robust to mutation model assumptions, these different dissimilarity measures should yield topologically similar UPGMA trees. This was not the case. We discovered that large differences are possible between UPGMA trees generated either by assuming IAM (all alleles are equally distant) or by assuming SMM (distance between alleles depends on their sizes). We also simulated populations of individual genotypes to compare the different kinds of dissimilarity measures.

## 2 MATERIALS AND METHODS

### 2.1 Mathematical methods

Most mutations at microsatellite loci increase or decrease repeat score by a single repeat unit. Therefore, a simple one‐step mutation model is usually assumed, although more complicated models have also been proposed (DiRienzo et al., 1994; Slatkin, 2002). The overall genetic dissimilarity between two individuals also depends on how similar the mutation process is between the SSR loci. In the first scenario, we assume a fixed mutation rate for all loci. Under such scenario, it is sufficient to explain allele size variation across loci by random distribution of mutational events among loci. However, a more realistic assumption is that mutation rate among loci is not fixed. Consequently, in the second scenario we assume the mutation rates to be variable. Under this scenario, the difference in the range of allele sizes is an intrinsic property of the locus, predetermined by locus‐specific mutation rates. We further consider and compare both scenarios.

Following steps are needed in developing metrics of dissimilarity between multilocus microsatellite genotypes for organisms of any ploidy:

1. Assess allelic dissimilarity between any two SSR alleles using an appropriate method.
2. Determine differences between SSR genotypes in a given locus (for di‐ and polyploids).
3. Determine dissimilarity between any two multilocus microsatellite genotypes by assuming constant and variable mutation rate at different SSR loci.

#### 2.1.1 Assessing allelic dissimilarity between SSR alleles

Given the SMM scenario, the first step is to measure differences between any two SSR alleles. Let *as_ij_* = *as*(*A_i_*) and *as_kj_* = *as*(*A_k_*) be allele sizes of two alleles *A_i_* and *A_k_* at the polymorphic locus *j*, respectively, and ltr*_j_* be the length of the tandem repeat unit at the locus *j*; then the difference between alleles *A_i_* and *A_k_* at that locus is calculated as $\Delta_{j}\left(A_{i},A_{k}\right)=\left|{\mathrm{as}}_{\mathrm{ij}}-{\mathrm{as}}_{\mathrm{kj}}\right|/{ltr}_{j}$, which is the difference in the number of tandem repeats between the two alleles. For example, if microsatellite consists of three nucleotides, ltr*_j_* = 3, and the recorded sizes of two alleles are *as_ij_* = 197 bp and *as_kj_* = 203 bp, then difference between these alleles equals 2. This can be considered an approximation of the number of mutation events (tandem repeat insertions or deletions that are not reversed) that result in transition of allele *A_i_* into allele *A_k_*. Even bearing in mind the reversed mutations (both increasing and decreasing in allele size), which of course may happen, the suggested difference between alleles increases with the actual number of mutations under assumption that the reversed mutations are randomly and evenly distributed across loci. In fact, when mutations that increase or decrease the size of the allele by one unit are equally likely, the stepwise mutation process can be described as a simple “random walk.” In a simple random walk, the distance travelled is proportional to the square root of steps (Codling, Plank, & S. Benhamou, 2008). In other words, in this case our approximation of number of mutation events becomes the squared difference in the number of tandem repeats. However, the theory of “random walk” refers to the expected value (average value of large number of repetitions), which works for a sample (population) as statistical estimation, but is not necessarily correct for each possible value (difference between two specific alleles). Moreover, the expected difference between alleles after *n* mutations should be of the order $\sqrt{n}$ for relatively large *n* (as *n* approaches infinity), although in study of closely related (recently diverged) populations small differences between SSR alleles may result from just a few mutation events. In addition, the TPM scenario of SSR evolution assumes that mutations of a few repeat units may occur, so that squaring difference in the number of tandem repeats may result in overestimating actual genetic distance between the corresponding SSR alleles. While using the squared differences between SSR alleles maybe well justified in phylogenetic analyses that are based on population estimates of allele differences with large number of mutations occurring over a long time interval with large number of repetitions, it is not clear whether squaring differences between alleles are a suitable for comparison of individual profiles. For example, it does not properly work in examples presented in “discussion.” Therefore, we will consider both the absolute and squared differences between SSR alleles.

Another question is whether the same difference between two pairs of SSR alleles at different loci contributes equally to dissimilarity between individuals. If the maximum number of tandem repeats (mutation events) varies from locus to locus, one can assume that the mutation rate is also variable and locus‐specific. For example, strong positive correlation between mutation rate and allele sizes has been shown by Xu, Peng, Fang, and Xu (2000) and Anmarkrud, Kleven, Bachmann, and Lifjeld (2008). Therefore, a particular difference $\Delta_{j}\left(A_{i},A_{k}\right)$ between two SSR alleles should have a greater impact on dissimilarity between individuals at loci where changes happen more slowly. In other words, when comparing to other loci, a larger maximum number of tandem repeats between alleles in locus *j*, $\Delta_{max}\left(j\right)$, suggests a higher mutation rate in that locus given the same evolutionary time among the compared loci. Assuming *T* is the time of divergence for a set of individuals (population) from a single common ancestor, the relative average time for one mutation in locus *j* equals $T_{j}=T/\Delta_{max}\left(j\right)$. For simplicity, we ignore back mutations here assuming that the number of such events is proportional to the number of insertions at each locus. Therefore,* T_j_* is actually proportional to the absolute average time for one mutation and can be used for measuring dissimilarity between SSR profiles. Using the term “time” below we mean “relative time.” Following these notions, a time difference between the two events of generating alleles *A_i_* and *A_k_* can be expressed as

(1) $$\tau_{j}\left(A_{i},A_{k}\right)=\Delta_{j}\left(A_{i},A_{k}\right)∙T_{j}=T∙\frac{\Delta_{j}\left(A_{i},A_{k}\right)}{\Delta_{max}\left(j\right)}$$

for $\Delta_{max}\left(j\right)=\left({max}_{j}-{min}_{j}\right)/{ltr}_{j}$, where max*_j_* and min*_j_* are the maximum and minimum allele sizes, respectively, detected at locus *j*. Therefore, since the time of divergence, *T*, is the same for all loci, the relative difference between two SSR alleles *A_i_* and *A_k_* at locus *j* can be estimated as

(2) $$\rho_{j}\left(A_{i},A_{k}\right)=\frac{\tau_{j}\left(A_{i},A_{k}\right)}{T}=\frac{\Delta_{j}\left(A_{i},A_{k}\right)}{\Delta_{max}\left(j\right)}=\frac{\left|{\mathrm{as}}_{\mathrm{ij}}-{\mathrm{as}}_{\mathrm{kj}}\right|/{\mathrm{ltr}}_{j}}{\left({max}_{j}-{min}_{j}\right)/{\mathrm{ltr}}_{j}}=\frac{\left|{\mathrm{as}}_{\mathrm{ij}}-{\mathrm{as}}_{\mathrm{kj}}\right|}{{max}_{j}-{min}_{j}}$$

with range $0\leq \rho_{j}\left(A_{i},A_{k}\right)\leq 1$. If back mutations are taken into account, then it would be reasonable also to consider the squared version of this difference between two SSR alleles:

(2′) $$\rho_{j}^{2}\left(A_{i},A_{k}\right)={\left[\frac{\Delta_{j}\left(A_{i},A_{k}\right)}{\Delta_{max}\left(j\right)}\right]}^{2}={\left[\frac{{\mathrm{as}}_{\mathrm{ij}}-{\mathrm{as}}_{\mathrm{kj}}}{{max}_{j}-{min}_{j}}\right]}^{2}.$$

#### 2.1.2 Determining differences between SSR genotypes in a given locus

The second step is to measure dissimilarity between two individuals at any given locus. This can be done using the approach suggested empirically by Bruvo et al. (2004, p. 2102, Equations 3 and 4) and in general algorithmic form by Kosman and Leonard (2005; p. 420, Equation (2)) with regard to *ρ*‐ or *ρ^2^*‐distance (Equation (2)) or (Equation 2′), respectively, between SSR alleles. The following explanations for *ρ*‐distances (Equation (2)) can be easily reformulated for *ρ^2^*‐distances (Equation 2′). Dissimilarity between two *q*‐ploid organisms A and B with alleles < *A_1_A_2_…A_q_* > and <*B_1_B_2_…B_q_* > at locus *j* is defined as follows. To each allele *A_i_*from one genotype, an allele *B_k_*from the second genotype is matched so as (a) to generate *q *different pairs of alleles where all alleles *A_i_*and *B_k_*are involved and each allele appears in just one pair and (b) to minimize the sum of *ρ*‐distances $\rho_{j}\left(A_{i},B_{k}\right)$ between *q *corresponding pairs of alleles. There are q!=1∙2∙3∙⋯∙q possibilities of the matching between alleles (for instance, for tetraploid *q = *4 and *q*! = 24). Finding the “best matches” (that delivers minimum of the sum of *ρ*‐distances in our case) is known as the “assignment problem” in operation research (Bellman, Cooke, & Lockett, 1970; Munkres, 1957). The distance between individuals A and B within the locus is determined as the minimum sum of *ρ*‐distances $\rho_{min}\left(A,B;j\right)$ derived for the best matches. This distance meets the parsimony principle, that is it expresses the minimum relative number of mutations (deletions or insertions of a tandem repeat) needed to get one genotype from another at locus *j* for A and B, which is proportional to the minimum time required for evolution of one individual into another. The normalized version of $\rho_{min}\left(A,B;j\right)$ (obtained by division by ploidy *q, *i.e., number of chromosome copies) is considered as the measure of dissimilarity between individuals A and B at locus *j*:

(3) $$d_{\mathrm{AB}}\left(j\right)=\frac{\rho_{min}\left(A,B;j\right)}{q},$$

so that it ranges from 0 to 1 and determines the minimum relative number of mutations per each copy of haploid genome at locus *j* for generating A from B—a kind of parsimony.

The following is dissimilarity between individuals A and B at locus *j* in the case of *ρ^2^*‐distances:

(3′) $${\overset{-}{d}}_{\mathrm{AB}}\left(j\right)=\frac{{\overset{-}{\rho }}_{min}\left(A,B;j\right)}{q},$$

where ${\overset{-}{\rho }}_{min}\left(A,B;j\right)$ is derived for *ρ^2^*‐distances (Equation 2′) as $\rho_{min}\left(A,B;j\right)$ for *ρ*‐distances (Equation (2)); ${0\leq \overset{-}{d}}_{\mathrm{AB}}\left(j\right)\leq 1$.

#### 2.1.3 Determining dissimilarity between any two multilocus microsatellite genotypes

Finally, the dissimilarities between two *q*‐ploid individuals *A* and *B* represented by their patterns at *n* microsatellite loci with regard to *ρ*‐ and *ρ^2^*‐distance are determined as follows:

(4) $$d_{\mathrm{AB}}^{v}=\frac{1}{n}∙\sum_{j=1}^{n}d_{\mathrm{AB}}\left(j\right)=\frac{1}{n∙q}∙\sum_{j=1}^{n}\rho_{min}\left(A,B;j\right),$$

(4′) $${\overset{-}{d}}_{\mathrm{AB}}^{v}=\frac{1}{n}∙\sum_{j=1}^{n}{\overset{-}{d}}_{\mathrm{AB}}\left(j\right)=\frac{1}{n∙q}∙\sum_{j=1}^{n}{\overset{-}{\rho }}_{min}\left(A,B;j\right),$$

with values in $\left[0,1\right]$ interval, where *v* designates variable rates of mutations at different loci. $d_{\mathrm{AB}}^{v}$ dissimilarity generalizes the measure of dissimilarity for haploid organisms (q=1) with SSR markers suggested by Ben‐David et al. (2016). Importantly, dissimilarities $d_{\mathrm{AB}}^{v}$ (Equation 4) and ${\overset{-}{d}}_{\mathrm{AB}}^{v}$ (Equation 4′) are obtained by assuming the parsimony principle and the stepwise mutation model with variable rates of mutations at different loci; scenario of these models is designated SMMv.

Assuming a constant rate of mutations at all loci and *ρ*‐distances (Equation (2)) between SSR alleles, another measure of dissimilarity $d_{\mathrm{AB}}^{c}$ between individuals A and B can be derived, where *c* designates a constant rate. In this case $T_{j}$ in Equation (1) is the same for all loci, so that the divergence time between two alleles *A_i_* and *A_k_* is proportional to $\Delta_{j}\left(A_{i},A_{k}\right)$ independently of locus *j*. Then $\Delta_{min}\left(A,B;j\right)$ is obtained as a solution of the corresponding “assignment problem” for differences $\Delta_{j}\left(A_{i},A_{k}\right)=\left|{\mathrm{as}}_{\mathrm{ij}}-{\mathrm{as}}_{\mathrm{kj}}\right|/{ltr}_{j}$ exactly the same way as $\rho_{min}\left(A,B;j\right)$ was derived for differences $\rho_{j}\left(A_{i},A_{k}\right)$ from Equation (2), and $0\leq \Delta_{min}\left(A,B;j\right)\leq q\Delta_{max}\left(j\right)$. Then

(5) $$d_{\mathrm{AB}}^{c}=\frac{1}{q}∙\frac{\sum_{j=1}^{n}\Delta_{min}\left(A,B;j\right)}{\sum_{j=1}^{n}\Delta_{max}\left(j\right)}$$

with values in $\left[0,1\right]$ interval.

Correspondingly, dissimilarity between individuals A and B can be determined assuming a constant rate of mutations at all loci and *ρ^2^*‐distances (Equation 2′) between SSR alleles:

(5′) $${\overset{-}{d}}_{\mathrm{AB}}^{c}=\frac{1}{q}∙\frac{\sum_{j=1}^{n}{\overset{-}{\Delta }}_{min}\left(A,B;j\right)}{\sum_{j=1}^{n}{\left[\Delta_{max}\left(j\right)\right]}^{2}}$$

with values in $\left[0,1\right]$ interval, where ${\overset{-}{\Delta }}_{min}\left(A,B;j\right)$ is calculated for squared values of $\Delta_{j}\left(A_{i},A_{k}\right)$ as $\Delta_{min}\left(A,B;j\right)$ for $\Delta_{j}\left(A_{i},A_{k}\right)$. Dissimilarities $d_{\mathrm{AB}}^{c}$ and ${\overset{-}{d}}_{\mathrm{AB}}^{c}$ are obtained by assuming the parsimony principle and the stepwise mutation model with a constant rate of mutations at all loci; we designate scenario of these models SMMc.

The suggested measures of dissimilarity between individuals can be interpreted as the minimum average time needed for transition of one randomly selected SSR allele at any locus of one individual into an arbitrary SSR allele of the second individual at the same locus under the assumption of variable and constant mutation rates at different loci. These metrics were developed using the stepwise mutation models under SMMv and SMMc scenarios, respectively, where dissimilarity between microsatellite alleles was calculated based on the allele sizes (*ρ*‐ or *ρ^2^*‐distance). This differs conceptually from the infinite alleles model IAM, where the binary difference is used, that is all different alleles are equally distant (see equation 2 in Kosman & Leonard, 2005). If all loci are polymorphic, then IAM dissimilarity of SSR genotypes between individuals A and B can be estimated as

(6) $$\delta_{\mathrm{AB}}=\frac{1}{q}∙\frac{\sum_{j=1}^{n}\delta_{min}\left(A,B;j\right)}{n},$$

where $\delta_{min}\left(A,B;j\right)$ is obtained as a solution of the corresponding “assignment problem” with regard to differences $\delta_{j}\left(A_{i},A_{k}\right)=1$ for any two different alleles $A_{i}\neq A_{k}$ (i≠k), and $\delta_{j}\left(A_{i},A_{i}\right)=0$ for all identical alleles.

Dissimilarities $d_{\mathrm{AB}}^{c}$ and ${\overset{-}{d}}_{\mathrm{AB}}^{c}$ (Equations 5 and 5′) are closely related to different measures

(7) $$d_{\mathrm{AB}}^{m}=\frac{1}{n∙q}∙\sum_{j=1}^{n}\Delta_{min}\left(A,B;j\right),$$

(7′) $${\overset{-}{d}}_{\mathrm{AB}}^{m}=\frac{1}{n∙q}∙\sum_{j=1}^{n}{\overset{-}{\Delta }}_{min}\left(A,B;j\right),$$

respectively, that simply equals minimum average number of mutations per each copy of haploid genome (MANMC) needed for generation of individual (A) from another individual (B) and vice versa.

Dissimilarity‐based approaches allow for effective data analyses in a case of missing records. Modifying equations for calculating dissimilarities between microsatellite genotypes with missing data are straightforward. One only needs to sum across all $n_{\mathrm{AB}}$ loci with available data for both individuals and substitute total number of loci n with $n_{\mathrm{AB}}$ ($n_{\mathrm{AB}}\leq n$) in Equations (4), (4′), (5), (5′), (6), (7).

### 2.2 Software

User‐friendly software LOCUS is freely available for computing dissimilarities between genotypes of haploid or diploid organisms obtained with dominant and codominant (including SSRs under assumption of IAM) molecular markers according to Kosman and Leonard (2005). The software can be downloaded at https://en-lifesci.tau.ac.il/profile/kosman. LOCUS also includes computational tools for calculating dissimilarities between microsatellite profiles developed in this paper (Equations 4, 4′, 5, 5′, 7, 7′) assuming SMM scenario. Data with missing records are permitted, and the corresponding dissimilarities can be calculated. In addition, the output includes basic information about a given data set with a number of descriptive parameters. LOCUS needs a programming environment of the Microsoft.NET Framework, which is an integral Windows component.

### 2.3 Simulations

We simulated populations of individual genotypes to compare the different kinds of dissimilarity measures when the mutation model, number of mutations differing between individuals, and time from ancestral state for each locus were known exactly. All simulations had a similar basic structure. We assigned an ancestral allele size to a locus (200 repeats) and simulated the evolution of the allele over time using a random walk process. For each generation, each locus had a probability to mutate to one step longer or to one step shorter drawn from a distribution with mean probability 0.25 and variance 0.02. Maximum probability for mutation event was 0.5. The simulation kept track of the present allele size, number of mutation events, and number of generations from the ancestor. For simulations with variable mutation rate per locus, we assigned each locus separately a mutation probability for each generation from the same random distribution as above.

We simulated both haploid clonal and haploid sexual genotypes. For haploid clonal lineages, eight loci were started simultaneously as a linked set of loci. Each had their independent mutation process over a same number of generations that was decided by the specific simulation. Sexual haploid lineages were assembled independently from eight single locus lineages that were each evolving a simulation specific, and usually a different number of generations from the ancestor.

All simulations were written with the R‐software (version 3.4.2). Code for the simulations is available in the supplement. The generated populations of genotypes were then further analyzed with LOCUS to compute alternative dissimilarity measures. We used the simulated data to evaluate goodness of fit of predicted genetic distances between genotypes using each dissimilarity measure ($d_{\mathrm{AB}}^{c}$, $d_{\mathrm{AB}}^{v}$, their “squared” versions ${\overset{-}{d}}_{\mathrm{AB}}^{c}{=\left(d_{\mathrm{AB}}^{c}\right)}^{2}$ and ${\overset{-}{d}}_{\mathrm{AB}}^{v}{=\left(d_{\mathrm{AB}}^{v}\right)}^{2}$ for haploids, and Bruvo's distance) to the known values of differences in number of generations and mutations. We estimated the fit using the root‐mean‐square error (RMSE), the coefficient of variation of the RMSE [CV(RMSE)], the mean absolute error (MAE), and the *R* ^2^ criteria (Table 1). RMSE and MAE are absolute measures of fit, while CV(RMSE) and *R* ^2^ are relative measures of fit.

**Table 1:** Summary of linear models where known relatedness between pairs of genotypes (either in mutation or generation number) is predicted with genetic dissimilarity measures under different scenarios of SSR evolution

Simulation attributes			Goodness of fit estimates			
Scenario	Difference between alleles		RMSE^c^	CV(RMSE)^d^	MAE^e^	*R* ^2^ f
	Predicted	Actual number of				
SMMc_1	$d_{\mathrm{AB}}^{c}$ a	Generations	0.092	0.304	0.075	0.925
	${\overset{-}{d}}_{\mathrm{AB}}^{c}$ a		0.041	0.366	0.030	0.921
	$d_{\mathrm{AB}}^{c}$	Mutations	0.094	0.311	0.077	0.921
	${\overset{-}{d}}_{\mathrm{AB}}^{c}$		0.043	0.377	0.031	0.915
SMMc_2	$d_{\mathrm{AB}}^{c}$	Generations	0.093	0.326	0.077	0.914
	${\overset{-}{d}}_{\mathrm{AB}}^{c}$		0.040	0.404	0.029	0.903
SMMv^b^	$d_{\mathrm{AB}}^{v}$ b	Generations	0.107	0.364	0.089	0.889
	${\overset{-}{d}}_{\mathrm{AB}}^{v}$ b		0.043	0.423	0.032	0.885
	$d_{\mathrm{AB}}^{v}$	Mutations	0.110	0.376	0.091	0.882
	${\overset{-}{d}}_{\mathrm{AB}}^{v}$		0.045	0.441	0.034	0.874

Note

Models were forced through zero intercept. SMMc_1 describes a stepwise mutation model simulation with constant mutation rate across loci after on average 691 generations of evolution (max = 1,362, min = 5); SMMc_2 respectively describes a stepwise mutation model simulation with constant rate of mutations across loci after on average 456 generations of evolution (max = 891, min = 2); SMMv describes a stepwise mutation model simulation with variable rate of mutations across loci after on average 254 generations of evolution (max = 518, min = 1). In each simulation a population of 100 individuals was sampled from a single haploid pedigree.

a Dissimilarities for the SMMc (Equations 5 and 5′).

b Dissimilarities for the SMMv (Equations 4 and 4′).

c Root‐mean‐square error (RMSE).

d Coefficient of variation of the RMSE.

e Mean absolute error (MAE).

f R‐square criterion.

### 2.4 Empirical data

We analyzed the following three data sets.

1. Bryozoans

*Cristatella mucedo* is a diploid freshwater bryozoan. For this study, we used data on eight microsatellite loci (Table 2) that were used to describe the genetic structure of *Cristatella* populations in Switzerland (Dünner, ETH‐Zurich, MSc‐thesis). Data were collected in 2012 hierarchically at different spatial scales. The data set consists of 197 *Cristatella* colonies from six large lakes. Collections were replicated within‐lakes by sampling several locations and within local patches by sampling several colonies per patch (Dünner, ETH‐Zurich, MSc‐thesis). Five of the used loci are described in Freeland, Jones, Noble, and Okamura (1999) (loci 1.1, 2.2, 2.9, 6.7, 9.4) the remaining three are unpublished.

- Wheat leaf rust

**Table 2:** SSR allele composition of *Cristatella mucedo* population (197 colonies)

Locus	Repeat size^a^	Missing data	Allele size^a^		Max difference between alleles^b^	Number of alleles	Proportion of homozygotes
			min	max			
1	2	0	197	229	16	9	0
2	2	4	242	270	14	8	0.28
3	2	1	207	309	51	7	0.08
4	2	0	102	194	46	12	0.22
5	3	0	188	221	11	7	0.06
6	2	0	194	208	7	7	0
7	2	0	244	254	5	4	0.22
8	2	0	154	208	27	11	0.02

a Number of nucleotides.

b Number of tandem repeats.

The data that we use consist of genotypes of single‐uredinial isolates of *Puccinia triticina *Eriks. (wheat leaf rust) collected from wheat in Russia in 2006–2014. Data analysis is based on eighteen microsatellite markers (Table 3; for details see Gultyaeva et al., 2017). In total, SSR genotypes of 192 isolates of wheat leaf rust were determined. *P. triticina *fungi are dikaryons where each cell is carrying two haploid nuclei. For using SSR markers this is similar to having a diploid organism.

- Wheat powdery mildew

**Table 3:** SSR allele composition of 192 isolates of *Puccinia triticina *Eriks

Locus	Repeat size^a^	Missing data	Allele size^a^		Max difference between alleles^b^	Number of alleles	Proportion of homozygotes
			min	max			
1	2	0	127	131	2	3	0.90
2	2	0	365	369	2	3	0.86
3	2	0	306	310	2	3	0.99
4	2	0	296	302	3	3	0.31
5	2	0	391	395	2	3	0.99
6	2	0	383	387	2	3	0.87
7	2	0	245	247	1	2	0.49
8	3	0	476	479	1	2	0.79
9	2	0	392	396	2	2	0.41
10	3	0	233	242	3	4	0.20
11	2	0	216	218	1	2	0.15
12	2	0	215	217	1	2	0.36
13	2	0	211	215	2	3	0.42
14	3	0	344	350	2	3	0.73
15	3	0	150	153	1	2	0.96
16	2	0	349	351	1	2	0.93
17	2	0	244	246	1	2	0.56
18	2	0	313	333	10	4	0.59

a Number of nucleotides.

b Number of tandem repeats.

A sample of *Blumeria graminis *f. sp. *tritici *(*Bgt*, wheat powdery mildew) isolates were collected from wild (*Triticum dicoccoides*) and domesticated (*Triticum aestivum* and *Triticum durum*) wheat species growing in Israel. Simple sequence repeats (SSR) alleles were determined for 57 isolates (19, 24 and 14 from *T. dicoccoides*, *T. aestivum*, and *T. durum*, respectively) with seven SSR markers (Table 4; for details see Ben‐David et al., 2016). *Bgt *fungi are monokaryons i.e., haploid.

**Table 4:** SSR allele composition of 57 isolates of *Blumeria graminis *f. sp. *tritici*

Locus	Repeat size^a^	Missing data	Allele size^a^		Max difference between alleles^b^	Number of alleles
			min	max		
1	3	14	155	509	118	28
2	4	5	276	284	2	3
3	2	1	180	202	11	11
4	3	4	243	303	20	10
5	4	1	153	165	3	4
6	4	3	192	260	17	10
7	3	4	266	560	98	27

a Number of nucleotides.

b Number of tandem repeats.

### 2.5 Data analysis

Dissimilarities $d_{\mathrm{AB}}^{v}$, $d_{\mathrm{AB}}^{c}$, $\delta_{\mathrm{AB}}$, and $d_{\mathrm{AB}}^{m}$ (Equations (4), (4′), (5), (5′), (6), (7), respectively) between individual SSR genotypes were calculated using LOCUS software (see above) for each dataset. We used the Mantel test (Mantel, 1967) to test the correlation of dissimilarity matrixes calculated with different measures for each pair of matrices for all three data sets. This allowed us to evaluate to which extent the different dissimilarity measures were in agreement when used on the same dataset. Mantel tests were calculated using the MXCOMP program of NTSYSpc package, version 2.2 (Exeter Software, Setauket, NY).

In addition, we tested the correspondence between clustering solutions obtained with the UPGMA dendrograms given the different models for measuring dissimilarity between SSR profiles. The UPGMA dendrograms with regard to each dissimilarity index were calculated using Mega7 (MEGA7: Molecular Evolutionary Genetics Analysis version 7.0 for bigger datasets (Kumar, Stecher, & Tamura, 2016)). For each dendrogram, the cophenetic ultrametric dissimilarities were calculated for all pairs of individuals (tips in a dendrogram) with COPH module of NTSYSpc package, version 2.2 (Exeter Software, Setauket, NY). When relevant, the goodness of fit for clustering with different dissimilarity matrices (matching the dendrogram structures derived with different models for comparison of SSR genotypes) was tested using the Mantel test (the MXCOMP program of NTSYSpc).

We visualized the differences between the UPGMA trees that were calculated based on different mutation model‐specific pairwise dissimilarities using “cophenoplot” function in r‐package “ape.” We also calculated the normalized symmetric difference (Robinson–Foulds distance) in the topology between the UMGMA trees using the r‐package “phangorn” (Schliep, 2011).

## 3 RESULTS

### 3.1 Simulations

The simulation results show that the Bruvo's distance between SSR alleles (equation 2 in Bruvo et al., 2004; Equation 8) does not express the corresponding actual differences between alleles in number of generations or mutations (Supporting information Figure S1). Correlations between the predicted distances and the actual differences between genotypes varied in a wide interval from 0.15 to 0.9 for the separate loci. Therefore, we simulated lineages assuming either variable or constant mutation rate and compared the average values of the predicted genetic distances across eight loci to the actual differences between the genotypes measured in number of generations or number of mutations.

Table 1 summarizes how well different dissimilarity measures ($d_{\mathrm{AB}}^{c}$, $d_{\mathrm{AB}}^{v}$ and their “squared” values ${\overset{-}{d}}_{\mathrm{AB}}^{c}{=\left(d_{\mathrm{AB}}^{c}\right)}^{2}$ and ${\overset{-}{d}}_{\mathrm{AB}}^{v}{=\left(d_{\mathrm{AB}}^{v}\right)}^{2}$, respectively) predict true distance between genotypes. We compared the relative performance of squared dissimilarity values to nonsquared ones in two scenarios where one hundred individuals were separated for a large number of generations (SSMc_1 average pairwise difference = 460 generations, max = 1,357, min = 1) or a fewer number of generations (SMMc_2 average pairwise difference = 286 generations, max = 892, min = 1). Qualitatively, the effect of “squaring” the dissimilarity measures was small. Root‐mean‐square error (RMSE) and mean absolute error (MAE) was always higher for nonsquared measures, suggesting poorer fit, but relative measures of fit CV(RMSE) and *R* ^2^ indicated better performance for the nonsquared measures (Table 1).

Using simulations, we also found that when we calculate the predicted distance of genotypes first assuming constant mutation rate and then use the same data assuming variable mutation rate, the two estimates are highly correlated. Interestingly, this was independent of the type of actual mutation rate used in the simulation. In other words, the two measures were highly correlated both when $d_{\mathrm{AB}}^{c}$ dissimilarity for constant mutation rate was applied to data where loci had a variable mutation rate, and vice versa when $d_{\mathrm{AB}}^{v}$ was applied to simulated data where loci had a constant mutation rate.

Genetic distance between the simulated haploid sexual SSR genotypes and difference in average age of those genotypes did not correlate. Age of the genotype was measured as the average number of generations the alleles of the loci were from the ancestor. We tested the correlation using a simulation where mutation rate of the loci was kept constant. We also considered a scenario where alleles of a genotype were nearly the same age. We simulated this by producing genotypes where the difference in number of generations from a common ancestor did not exceed nine. However, we found the same result—no correlation between genetic distance and difference in average age of the genotypes. We then generated groups of genotypes of nearly the same age with a fixed average difference in age between the groups. More specifically, we generated 100 groups with 20 genotypes at each, setting the age difference to 15 generations between the successive groups. We then calculated pairwise distances of average differences between groups (DAD; Kosman & Leonard, 2007; Kosman, 2014) using $d_{\mathrm{AB}}^{c}$ and ${\overset{-}{d}}_{\mathrm{AB}}^{c}$ dissimilarities and compared with the corresponding differences in the age of the groups (multiples of 15). The predicted relatedness of genotypes based on the $d_{\mathrm{AB}}^{c}$ distance was much stronger than that for the ${\overset{-}{d}}_{\mathrm{AB}}^{c}$ distance (*R* ^2^ = 0.873 vs. 0.403, and CV(RMSE) = 0.399 vs. 0.973; Supporting information Figure S2).

We discovered a similar relationships between genetic distance and age for haploid clonal genotypes with the $d_{\mathrm{AB}}^{c}$ and $d_{\mathrm{AB}}^{m}$ dissimilarities, which is rather expected due to resemblance of the definitions (Equations 5 and 7). This result was independent of whether the simulation scenario was based on either constant (SMMc) or variable (SMMv) mutation rates at different loci.

### 3.2 Bryozoans

We genotyped 197 colonies of *Cristatella mucedo* for 8 microsatellite loci (see Table 2 for overview of loci). Allele differences were large across the loci varying from 5 to 51 repeat units (Table 2). Two loci had less variation in allele differences (five and seven repeat units), four loci were in the moderate range (11–27 repeat unit differences), and two loci had large difference in repeat numbers (46 and 51). Only 0.3% of genotype data were missing (five individuals had a genotype where data for one locus was missing). We calculated the dissimilarities $d_{\mathrm{AB}}^{v}$, $d_{\mathrm{AB}}^{c}$, $\delta_{\mathrm{AB}},$ and $d_{\mathrm{AB}}^{m}$ between individual genotypes using Equations (4), (4′), (5), (5′), (6), (7), respectively and adjusted for missing data (see Discussion) with n≤8 (number of loci with available data for both genotypes in each pairwise comparison) and q=2 because *Cristatella* is diploid.

Correlations between different dissimilarity matrixes varied from low to high (0.374–0.931) (Mantel tests, Table 5a, below diagonal). We also found statistically significant correlations between cophenetic ultrametric distance matrixes generated from the corresponding UPGMA dendrograms (Table 5a, above diagonal values). Nearly absolute correlation between dissimilarities obtained with $d_{\mathrm{AB}}^{c}$ and $d_{\mathrm{AB}}^{m}$ is expected from their definitions (Equations 5 and 7) when just a few data are missing. Except for this, we found the highest correlation between dissimilarities $\delta_{\mathrm{AB}}$ and $d_{\mathrm{AB}}^{v}$ for IAM and SMM with variable mutation rate (0.896) and the corresponding cophenetic ultrametric distances (0.931). Correlation between dissimilarities $d_{\mathrm{AB}}^{v}$ and $d_{\mathrm{AB}}^{c}$ for SMMv and SMMc, respectively, was also relatively high (0.814), though association between the corresponding cophenetic distances was much weaker (0.505). Apart from the results for IAM and SMMv, all correlations between the original matrices were larger than those for the corresponding ultrametric distances.

**Table 5:** Association between original dissimilarity matrixes (below diagonal) and cophenetic ultrametric distances for UPGMA dendrograms obtained with the corresponding dissimilarities (above diagonal) measured with Mantel tests for (a) *Cristatella mucedo* population; (b) collection of *Puccinia triticina* isolates; and (c) collection of *Blumeria graminis* isolates

	IAM	MANMC	SMMc	SMMv
(a)				
IAM		0.374	0.375	0.931
MANMC	0.59		0.999	0.504
SMMc	0.591	0.999		0.505
SMMv	0.896	0.814	0.814	
(b)				
IAM		0.729	0.728	0.818
MANMC	0.766		1	0.665
SMMc	0.766	1		0.665
SMMv	0.954	0.805	0.805	
(c)				
IAM		0.229	0.253	0.508
MANMC	0.401		0.847	0.641
SMMc	0.309	0.876		0.718
SMMv	0.616	0.698	0.765	

Note

IAM: $\delta_{\mathrm{AB}}$ dissimilarity for the infinite alleles model (Equation 6); MANMC: $d_{\mathrm{AB}}^{m}$ dissimilarity (minimum average number of mutations per a copy of haploid genome; Equation 7); SMMc: $d_{\mathrm{AB}}^{c}$ dissimilarity for the stepwise mutation model with a constant rate of mutations (Equation 5); SMMv: $d_{\mathrm{AB}}^{v}$ dissimilarity for the stepwise mutation model with a variable rate of mutations (Equation 4).

**Figure 1:**
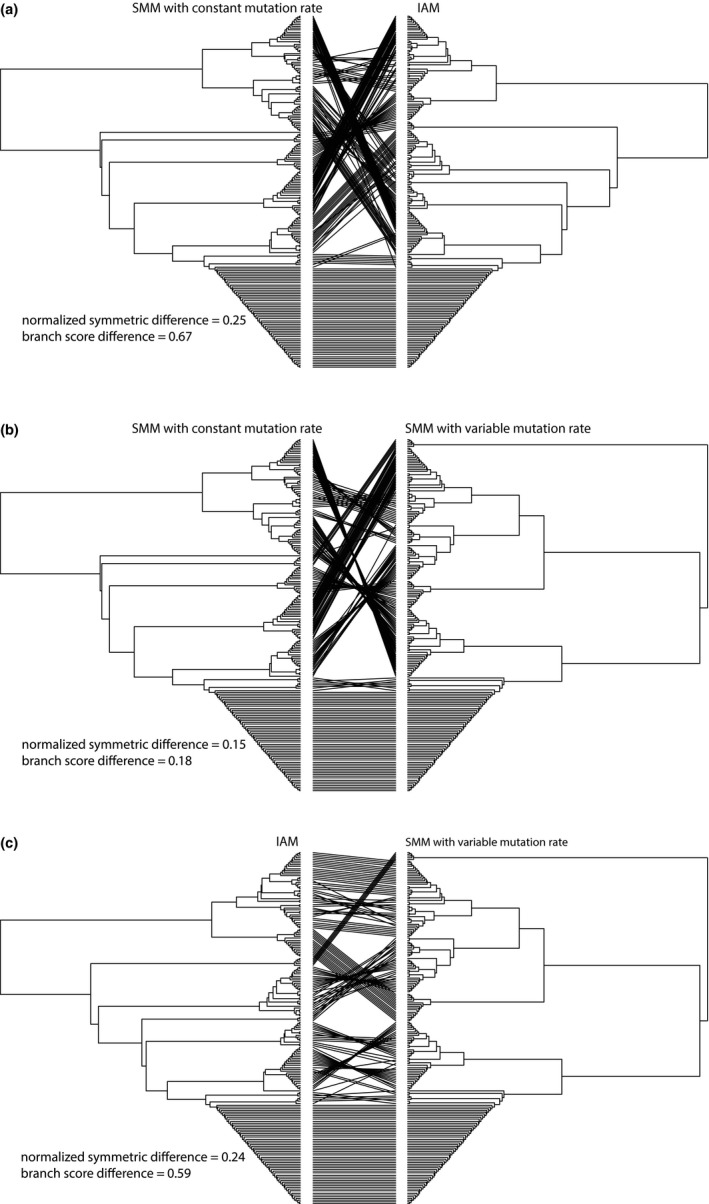
Comparison of UPGMA trees calculated for the *Cristatella mucedo* dataset using different pairwise dissimilarity matrices. Normalized symmetric difference (Robinson–Foulds distance) reports the proportion of partitions that are not shared between the trees, while the Branch Score Difference is a measure of branch length differences between the two trees (Steel & Penny, 1993). (a) Comparison between UPGMA trees calculated assuming SSM with constant mutation rate ($d_{\mathrm{AB}}^{c}$) and IAM ($\delta_{\mathrm{AB}}$). (b) Comparison between UPGMA trees calculated assuming SSM with constant mutation rate ($d_{\mathrm{AB}}^{c}$) and SSM with variable mutation rate ($d_{\mathrm{AB}}^{v}$). (c) Comparison between UPGMA trees calculated assuming SSM with variable mutation rate ($d_{\mathrm{AB}}^{v}$) and IAM ($\delta_{\mathrm{AB}}$)

Comparison of the topology of UPGMA trees revealed large differences between trees that were derived assuming different mutation models (Figure 1). Differences in the topology generated assuming the IAM scenario versus those for SMM with constant and variable mutation rate were 25% and 24%, respectively, while the difference between topologies of the two SMM trees was 15% (Figure 1).

### 3.3 Wheat leaf rust

We had genotype data for 192 *P. triticina* isolates covering 18 microsatellite loci (see Table 3 for overview of data). Differences between the SSR alleles were generally very small (1–3 repeat units) with the exception at one locus where the alleles were 10 repeat units apart. There were no missing data. We calculated four different types of dissimilarities $d_{\mathrm{AB}}^{v}$, $d_{\mathrm{AB}}^{c}$, $\delta_{AB,}$ and $d_{\mathrm{AB}}^{m}$ between individual genotypes using Equations (4), (4′), (5), (5′), (6), (7), respectively, for n=18 (number of loci) and q=2.

Comparison of the resulting dissimilarity matrixes using Mantel's tests showed that the matrixes correlated statistically significantly, but the correlations differed in magnitude (Table 5b, below diagonal values). Similar analysis comparing UPGMA derived cophenetic ultrametric distances is presented in Table 5b (above diagonal values). We found a very strong correlation (*r* = 0.954) between dissimilarity matrixes calculated with $\delta_{\mathrm{AB}}$ and $d_{\mathrm{AB}}^{v}$ measures, while the correlation between corresponding cophenetic distances was weaker, but still high (*r* = 0.818). All other correlations were of moderate level, and the estimates obtained for the original matrices were larger than those for the corresponding ultrametric distances.

As expected from definitions (Equations 5 and 7) in the case of no missing data, the dissimilarities obtained with $d_{\mathrm{AB}}^{c}$ and $d_{\mathrm{AB}}^{m}$ were absolutely correlated. Consequently, the corresponding matrices of cophenetic distances are also in total agreement.

**Figure 2:**
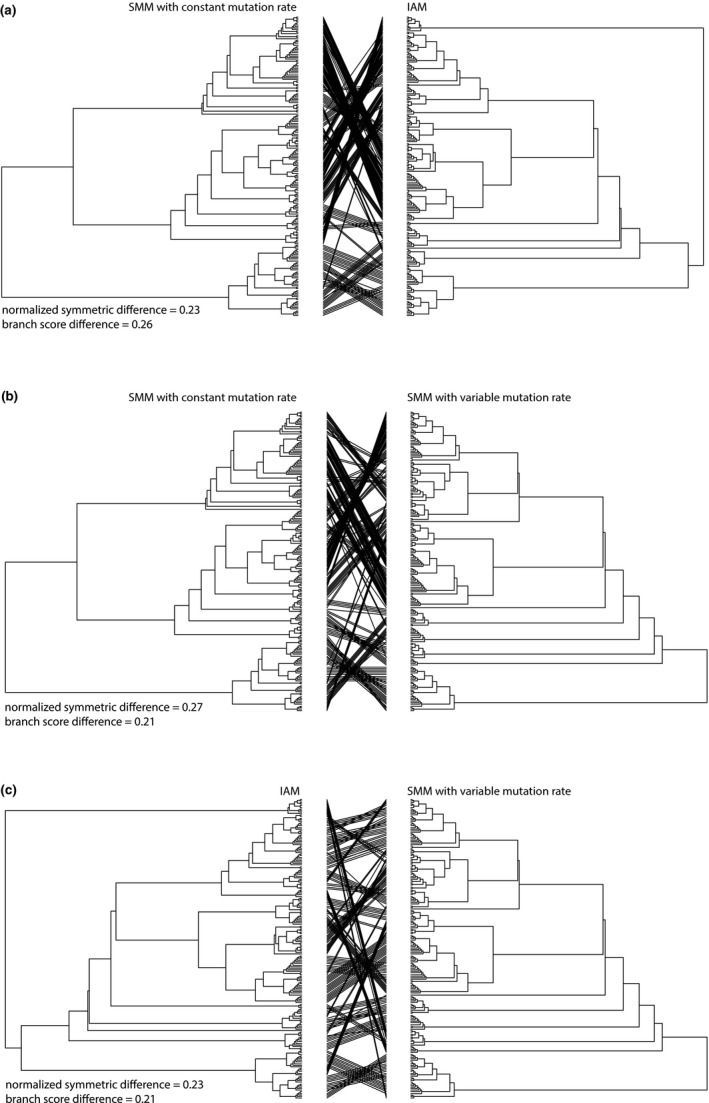
Comparison of UPGMA trees calculated for the leaf rust (*Puccinia triticina*) dataset using different pairwise dissimilarity matrices. Normalized symmetric difference (Robinson–Foulds distance) reports the proportion of partitions that are not shared between the trees, while the Branch Score Difference is a measure of branch length differences between the two trees (Steel & Penny, 1993). (a) Comparison between UPGMA trees calculated assuming SSM with constant mutation rate ($d_{\mathrm{AB}}^{c}$) and IAM ($\delta_{\mathrm{AB}}$). (b) Comparison between UPGMA trees calculated assuming SSM with constant mutation rate ($d_{\mathrm{AB}}^{c}$) and SSM with variable mutation rate ($d_{\mathrm{AB}}^{v}$). (c) Comparison between UPGMA trees calculated assuming SSM with variable mutation rate ($d_{\mathrm{AB}}^{v}$) and IAM ($\delta_{\mathrm{AB}}$)

Comparison of the topology of UPGMA trees revealed large differences between trees derived assuming different mutation models (Figure 2). Differences between IAM and SMM with constant and variable mutation rate based UPGMA topologies were 23% for both comparisons, while the difference between topologies of the two SMM trees was 27% (Figure 2).

### 3.4 Wheat powdery mildew

We had genotype data of 57 *B. graminis* isolates from wild and domesticated wheats in 7 microsatellite loci (see Table 4 for overview). Differences between the SSR alleles were very large (2–118 repeat units). Two loci had small differences between alleles (2 and 3 repeat units), three loci had moderate differences (11–20 repeat units), and two loci had very large differences in repeat numbers (98, and 118). Missing data were common (among 8% of genotypes in total, about 40% were in locus #1). We calculated the different types of dissimilarities $d_{\mathrm{AB}}^{v}$, $d_{\mathrm{AB}}^{c}$, $\delta_{AB,}$ and $d_{\mathrm{AB}}^{m}$ between individual genotypes using Equations (4), (4′), (5), (5′), (6), (7), respectively, adjusting for missing data (see Discussion) with n≤7 (number of loci with available data for both genotypes in each pairwise comparison) and q=1 because *B. graminis* is monokaryotic fungi (equivalent to haploid).

Correlations between all dissimilarity matrixes were statistically significant (Table 5c). The strength of correlation coefficients varied from low to moderate (0.229–0.765). Only the correlation between $d_{\mathrm{AB}}^{c}$ and $d_{\mathrm{AB}}^{m}$ dissimilarities was high (*r* = 0.876) as was for the corresponding cophenetic distances (0.847). Except for this, the results obtained with SMM for variable and constant mutation rates were the most qualitatively similar with correlations 0.765 and 0.718 for original dissimilarities $d_{\mathrm{AB}}^{v}$ and $d_{\mathrm{AB}}^{c}$ and cophenetic distances, respectively. All estimates of association between the original matrices were larger than those for the corresponding ultrametric distances.

**Figure 3:**
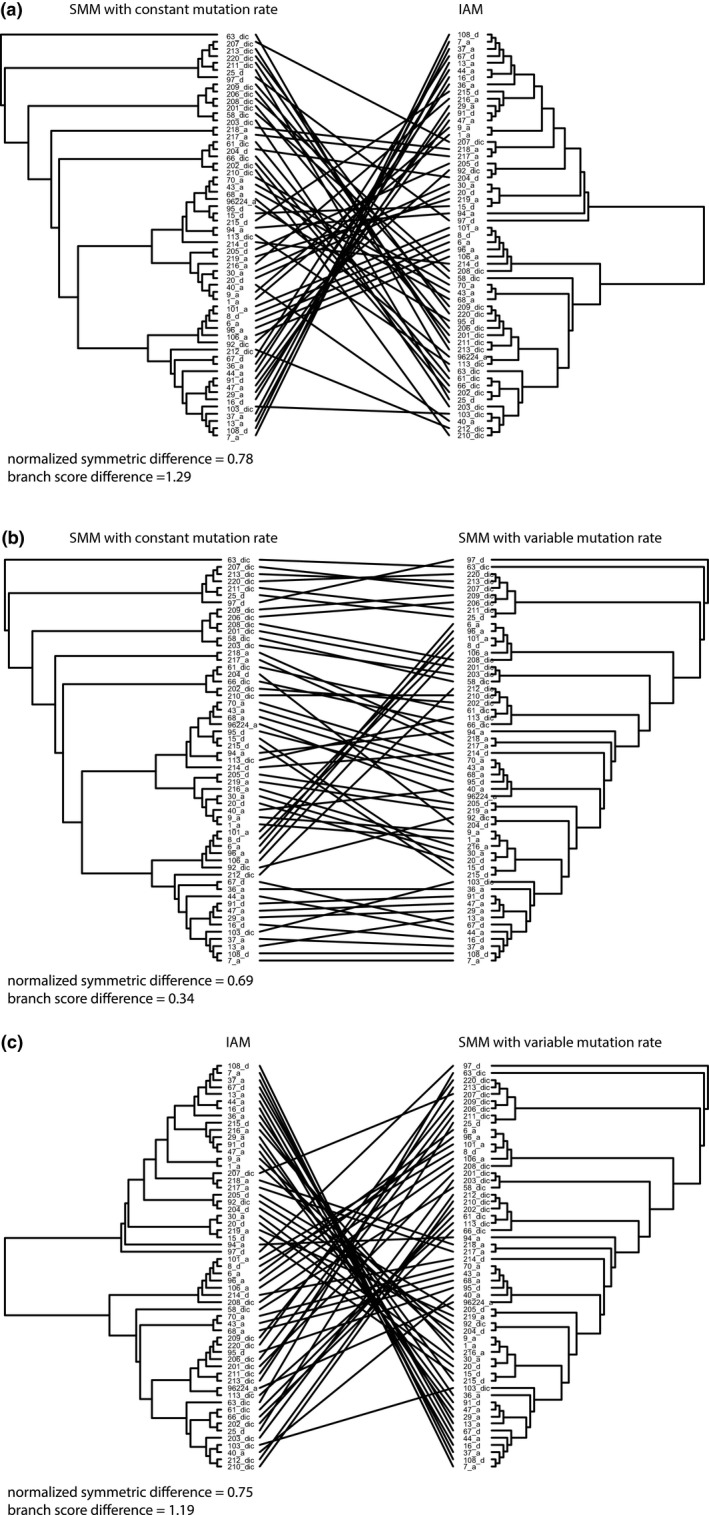
Comparison of UPGMA trees calculated for the powdery mildew (*Blumeria graminis*) dataset using different pairwise dissimilarity matrices. Normalized symmetric difference (Robinson–Foulds distance) reports the proportion of partitions that are not shared between the trees, while the Branch Score Difference is a measure of branch length differences between the two trees (Steel & Penny, 1993). (a) Comparison between UPGMA trees calculated assuming SSM with constant mutation rate ($d_{\mathrm{AB}}^{c}$) and IAM ($\delta_{\mathrm{AB}}$). (b) Comparison between UPGMA trees calculated assuming SSM with constant mutation rate ($d_{\mathrm{AB}}^{c}$) and SSM with variable mutation rate ($d_{\mathrm{AB}}^{v}$). (c) Comparison between UPGMA trees calculated assuming SSM with variable mutation rate ($d_{\mathrm{AB}}^{v}$) and IAM ($\delta_{\mathrm{AB}}$)

The resulting UPGMA topologies were highly affected by the assumed mutation model (Figure 3). Topology of IAM‐based UPGMA tree differed from both SMM trees by >75% (Figure 3). Also, the two SMM‐based trees differed by 69% (Figure 3), indicating how profound effect the choice of dissimilarity metric had on the resulting topology.

Our results using empirical data sets (Supporting information Table S1) demonstrate that the mode of SSR evolution (constant or variable mutation rate) has a much larger influence on inferred relationship between individuals than the way of measuring the evolutionary difference between SSR alleles (absolute vs. squared difference). We found that the two dissimilarity measures assuming either constant or variable mutation rates differed by a large effect when analyzing real data, while for the simulated data the difference was minor (see above).

## 4 DISCUSSION

We propose new metrics for measuring dissimilarity between SSR genotypes because the existing ones do not seem to work properly as explained in the next two sections. We compare and discuss the utility of the newly suggested approaches for analyzing variation within and among populations.

### 4.1 Allele size‐based distance measures developed for populations of diploid organisms do not properly work when adjusted to individuals

Two distance measures for comparison between diploid populations on the basis of microsatellite data were developed by Goldstein, Linares, Cavalli‐Sforza, and Feldman (1995a), Goldstein, Linares, Cavalli‐Sforza, and Feldman (1995b): the average squared distance *D_1_* (ASD method) and the squared difference between the means of allele size in two populations (*δμ*)^2^ (SMD method). Slatkin (1995) suggested a measure of differentiation among populations, *R*_ST_. Since a single individual can formally be considered as population that consists of one entity, these indices were also mechanistically used for comparison of individuals (for instance, see Udupa et al., 1999). The following example demonstrates shortcomings of applications *D_1_*, (*δμ*)^2^, and *R*_ST_ to measuring dissimilarity between SSR genotypes.

Let us consider profiles of four diploid individuals at a single SSR locus with a repeating motif consisting of two nucleotide bases and primer length of 19 nucleotides: $i_{1}=\left(41,53\right)$; $i_{2}=\left(43,51\right)$; $i_{3}=\left(45,49\right)$; and $i_{4}=\left(43,51\right)$. Then, in terms of numbers of repeat motifs (actual allele sizes): $i_{1}=\left(11,17\right)$; $i_{2}=\left(12,16\right)$; $i_{3}=\left(13,15\right)$; and $i_{4}=\left(12,16\right)$. The mean allele sizes for all individuals are equal: $\mu_{1}=\left(11+17\right)/2=14$, $\mu_{2}=\left(12+16\right)/2=14$, $\mu_{3}=\left(13+15\right)/2=14$, and $\mu_{4}=\left(12+16\right)/2=14$. Therefore, according to (*δμ*)^2^, these individuals are interpreted as “identical” because ${\left(\mu_{k}-\mu_{j}\right)}^{2}={\left(14-14\right)}^{2}=0$ for *k*, *j = 1,2,3,4*. Slatkin's differentiation coefficient *R*_ST_ does not distinguish between the second and third individuals because $R_{ST}\left(i_{2},i_{3}\right)=0$ either. On the other hand,

$$D_{1}\left(i_{2},i_{4}\right)={\left(12-12\right)}^{2}∙\frac{1}{2}∙\frac{1}{2}+{\left(12-16\right)}^{2}∙\frac{1}{2}∙\frac{1}{2}+{\left(16-12\right)}^{2}∙\frac{1}{2}∙\frac{1}{2}+{\left(16-16\right)}^{2}∙\frac{1}{2}∙\frac{1}{2}=8,$$

that is two individuals $i_{2}$ and $i_{4}$ with identical profiles are interpreted as different according to *D_1_*. Thus, neither (*δμ*)^2^ and *D_1_* distances nor $R_{ST}$ coefficient of differentiation are relevant since they distort actual relationships between individuals.

### 4.2 Bruvo's distance is not suitable for measuring difference between SSR alleles

New measures of dissimilarity between microsatellite genotypes were developed assuming that distance between microsatellite alleles is associated with the difference between sizes (number of tandem repeats) of those alleles (Equations (2) and 2′). This idea was first realized by Bruvo et al. (2004), although they did not raise it directly or address in detail in their study.

Bruvo's distance between SSR alleles (Bruvo et al., 2004) was suggested as a consequence of the generalized stepwise mutation model (SMM), in which mutations may result in an increase or decrease by any finite number of repeat units (Slatkin, 2002). The Bruvo's distance between two SSR alleles with differences *k* in the number of repeat units was determined as

(8) $$d_{a}=1-2^{-\left|k\right|}$$

(equation 2 in Bruvo et al., 2004; see Appendix S1 for details). The idea of measuring dissimilarity between microsatellite alleles with nonlinear functions of the corresponding differences in allele sizes can be further developed (see Appendix S1). However, Bruvo's distance does not properly express actual differences between SSR loci (shown by simulations; Supporting information Figure S1) mainly because it almost immediately approaches its maximum value even for relatively small differences between alleles. Effectively, differences in four and more repeat sizes make the alleles “absolutely” different. Moreover, this means that any two alleles with differences of more than five repeat sizes from a given allele are nearly equally maximally distant from the latter one, that is sensitivity of the Bruvo's distance is very low.

### 4.3 Model comparisons—simulations

Simulation results under the SMM scenario clearly demonstrated that the commonly used Bruvo's distance between SSR alleles is inappropriate when differences between allele sizes exceed five tandem repeats (Supporting information Figure S1). Simulations in general proved powerful for examining the sensitivity of the proposed metrics and their interpretation with respect to different evolutionary scenarios of SSR loci. Especially, valuable is the opportunity to relate the differences in allele sizes to variation in true relatedness among individuals. Since comparison of populations is usually based on the squared differences between alleles, we also analyzed the same metrics with regard to the squared differences. The results of our simulations suggest that the predictive power of the dissimilarity measures that are based on the absolute differences between allele sizes is generally stronger or equal to that based on the squared differences.

### 4.4 Model comparisons—experimental data

Following the results of simulations, only the newly developed metrics that base on the absolute differences between allele sizes (Equation (2)) were compared with real data. The four different types of dissimilarities $d_{\mathrm{AB}}^{v}$, $d_{\mathrm{AB}}^{c}$, $\delta_{\mathrm{AB}},$ and $d_{\mathrm{AB}}^{m}$ between individual SSR genotypes (Equations (4), (4′), (5), (5′), (6), (7), respectively) correspond to the three models of microsatellite evolution: IAM ($\delta_{\mathrm{AB}}$), SMMc ($d_{\mathrm{AB}}^{c}$ and $d_{\mathrm{AB}}^{m}$), and SMMv ($d_{\mathrm{AB}}^{v}$). In the case of no missing data, two dissimilarities $d_{\mathrm{AB}}^{c}$ and $d_{\mathrm{AB}}^{m}$ are totally correlated providing absolutely congruent solutions of all research problems based on manipulations with dissimilarity matrices (e.g., clustering, ordination, diversity analyses etc.). However, increasing amount of missing identifications of SSR alleles may lead to discrepancy in results obtained with the two dissimilarities related to SMMc (see analysis of wheat powdery mildew isolates, Table 5c). Therefore, assuming SMMc scenario, we would recommend using $d_{\mathrm{AB}}^{c}$ dissimilarity, which is less sensitive for missing data. So, we will further compare only three dissimilarities $d_{\mathrm{AB}}^{v}$, $d_{\mathrm{AB}}^{c}$, and $\delta_{\mathrm{AB}}$, one for each of the three models of microsatellite evolution.

In our analysis of the three empirical data sets, we found that different model‐dependent approaches to measuring dissimilarity may generally lead to inconsistent description of the relationships between SSR genotypes in both the original measures and those derived from the UPGMA dendrograms (Table 5). Except for one case (bryozoans with dissimilarities related to IAM and SMMv), correlations were usually lower for cophenetic ultrametric distances than for corresponding original dissimilarities based on each of the considered models. This means that absence of absolute correlation between original dissimilarities for different models has probably even stronger effect on disagreement in relationships between genotypes as displayed in the corresponding structured forms shaped by a clustering method (e.g., UPGMA dendrogram in our case).

Even if two original dissimilarity matrices correlate from moderate to high extent, further analyses based on those dissimilarities may describe the system in question incongruently and result in contradictory conclusions. The empirical data we analyzed did not provide any clear indication on which models and dissimilarities deliver the most compatible outcomes with matching conclusions. For example, the most correlated dissimilarities for bryozoan and leaf rust genotypes were $d_{\mathrm{AB}}^{v}$ and $\delta_{\mathrm{AB}}$ for SMMv and IAM scenarios of microsatellite evolution. On the other hand, for powdery mildew SSR genotypes the highest association was between $d_{\mathrm{AB}}^{v}$ and $d_{\mathrm{AB}}^{c}$ dissimilarities for two stepwise mutation models with variable and constant mutation rate. We found relatively large topological differences in the UPGMA trees calculated using different dissimilarity measures for SSR genotypes (Figures 1, 2, 3), suggesting that assumptions of the underlying mutation model have significant consequences for inferences on genetic structure within sampled individuals. This is a somewhat problematic finding as such use of SSR markers is common in ecological population genetics. Since results and inferences obtained with different models are not generally consistent, selection of a theoretically suitable dissimilarity measure becomes a key issue in performing adequate and valid dissimilarity‐based analyses. However, simple solution does not seem possible because mode of SSRs evolution and hidden subdivision of individuals into groups within natural populations are generally unknown. Therefore, we suggest exploratory analyses of genetic relationships between sampled individuals using a few relevant methods for SSR profiles (e.g., dissimilarities for IAM, SMMc, and SMMv mutation models, and SMM with Bruvo's distance between SSR alleles) to formulate hypotheses about the structure of individuals within the sample on the basis of each method. The suggested hypotheses can be further tested either with logically consistent tools of population genetics (e.g., differentiation among putative groups of individuals), or biological experiments to determine and justify well interpretable population subdivision.

### 4.5 Relationship among populations

Despite comparison of populations is beyond the main objective of our study, we comment on the possibility for a hidden link between diversity within and among populations, and dissimilarity between individuals.

Both microsatellite mutation models are relevant for the two different approaches that are commonly used for analyzing genetic diversity and structure of populations with SSR markers. The first common use of SSR data is to simply count alleles and their frequencies at each locus, calculating within locus statistics independently of other loci, usually averaging the corresponding statistics across all loci. The second common use of SSR data is to measure dissimilarity between SSR profiles of individuals across loci, and use the attained matrix of pairwise dissimilarities for exploratory analysis (e.g., clustering, ordination) and/or assessment of population characteristics (Excoffier, Smouse, & Quattro, 1992; Kosman, 2014; Kosman & Leonard, 2007). Dealing with the entire multilocus, individual patterns for calculating dissimilarities between individuals presumes that associations among alleles at different loci are taken into account, in contrast to the allele frequency approach. We call these two approaches “allele frequency” and “dissimilarity” methods, respectively. The latter methods can be subdivided into two different groups—those based on “averaging” and those based on “assignment” (Kosman, 2014; Kosman & Leonard, 2007). Remarkably, some allele frequency and average‐based dissimilarity methods can be identical (e.g., Kosman, 2003), so there is no absolute separation between them.

One important distinction between the allele frequency versus dissimilarity methods is the use of information about proximity between different SSR alleles determined in terms of allele sizes. With the allele frequency methods, all alleles are implicitly considered as equally distant. This means that the data are analyzed assuming infinite alleles model (IAM), that is any mutation of one allele into any other one is equally probable. The stepwise mutation model (SMM) and two‐phase model (TPM) assumes that mutations are more likely between SSR alleles that are closer in size. Therefore, considering degree of difference between alleles (proximity of the corresponding allele sizes) in dissimilarity‐based approaches may improve resolution and accuracy of data analysis. Yet, dissimilarity‐based approaches can also be implemented when only identity of alleles is considered. Thus, a suitable dissimilarity measure may yield valid applications under assumptions of SSR evolution scenarios.

Dissimilarity‐based approaches can be effectively used in a case of missing data, which is a common problem when using multilocus genotyping with molecular markers (Schluter & Harris, 2006). Missing data are usually dealt with either by eliminating loci or individuals with missing data, or by imputing values to replace the missing ones. Imputing is done according to a special algorithms and statistical properties of the given data set. Cutting loci or individuals leads to loss of data, which can be significant, while in the case of imputing data the issue of valid interpretation of the results necessarily raises because of some uncertainty in analyzing the partially fictive data. Fortunately, dissimilarity‐based approaches avoid such problems. If dissimilarity between genotypes is defined as “average” across loci (that is always possible), then for a given pair of individuals it can be calculated using the data that are available for those individuals. One needs to omit records only for affected loci of one of the individuals compared and just for the considered pair of genotypes. This effectively uses almost all the available information in the original data and analyses with simulated data are not necessary.

## 5 CONCLUSIONS

We derived new dissimilarity measures for microsatellite profiles of haploid, diploid, and polyploid organisms assuming different basic models of SSR allele evolution. Goodness of fit of these measures for determining actual relatedness among SSR genotypes versus their squared versions and the most commonly used Bruvo's distance was evaluated using simulations. It was shown that (a) the Bruvo's distance is not generally suitable for proper analyses of SSR genotypes under assumption of the SMM scenario; (b) the newly developed measures based on simple differences between allele sizes provide more accurate assessments of relationships between SSR genotypes as compared with the measures based on the squared differences between alleles. We also demonstrated that commonly used measures of distance between populations assuming SSR evolution according to the SMM cannot be applied to comparison between genotypes because they distort actual relationships among them. We then evaluated the performance of the new measures using data from real populations. We conclude that these measures facilitate discovery of initial structure within a set of individuals and seem the only way to provide reasonable alternatives for establishing putative relationships among individuals in natural populations using microsatellite data. The new dissimilarity‐based metrics are also suitable for analyzing diversity within and among originally predetermined populations.

## DATA ARCHIVING

Genotype data used in the examples of bryozoans *Cristatella mucedo*, wheat pathogens *Puccinia triticina *Eriks., and *Blumeria graminis *f. sp. *tritici* are available from the Dryad Digital Repository: https://doi.org/10.5061/dryad.1b8n2b4 (Data files: cristatella_mucedo_kosman_jokela, SSRs_Puccinia_triticana_Russia, and Blumeria_graminis_57_isolates_SSR_data_7_loci, respectively).

## CONFLICT OF INTEREST

The authors declare no conflict of interest.

## AUTHORS' CONTRIBUTION

E.K. and J.J. conceived the study; E.K. developed the models and computational tools; J.J. provided a part of data and performed simulations; E.K. and J.J. analyzed and interpreted the data, and wrote the manuscript.
