# Supplementary figures and images for: Dissimilarity of individual microsatellite profiles under different mutation models: Empirical approach
Source: Ecol Evol. 2019 Mar 19;9(7):4038–54. doi: 10.1002/ece3.5032 (PMC6467862; doi:10.1002/ece3.5032)

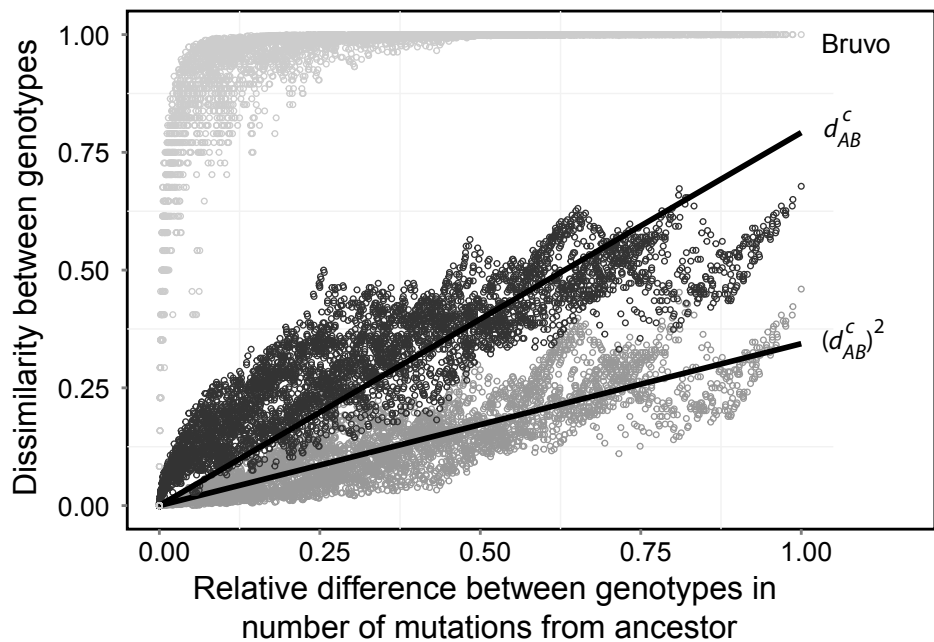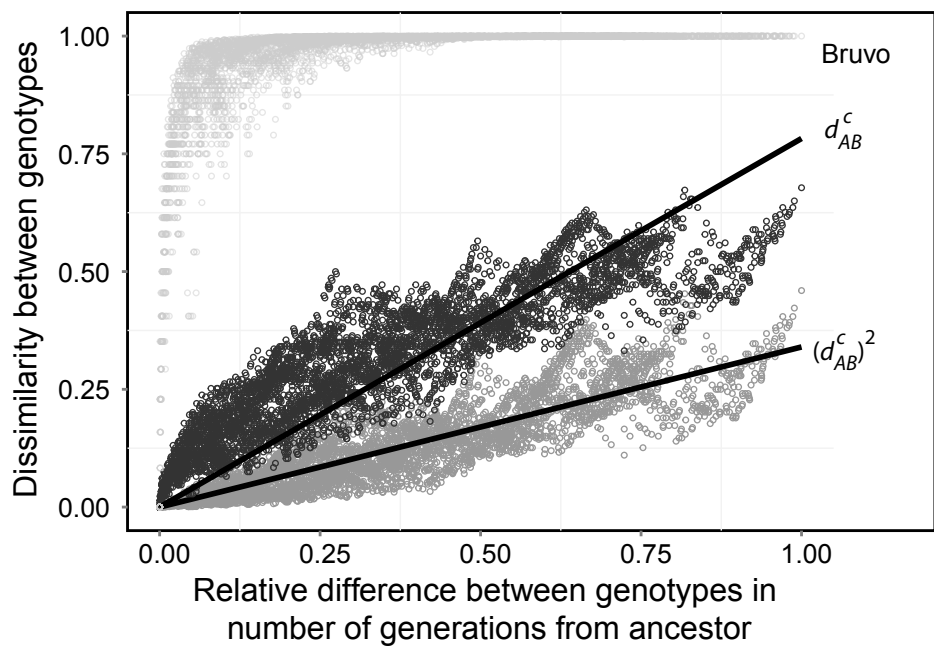

Figure 1S. Kosman and Jokela

Supplement: Supplementary file 1 [file ECE3-9-4038-s001.pdf]

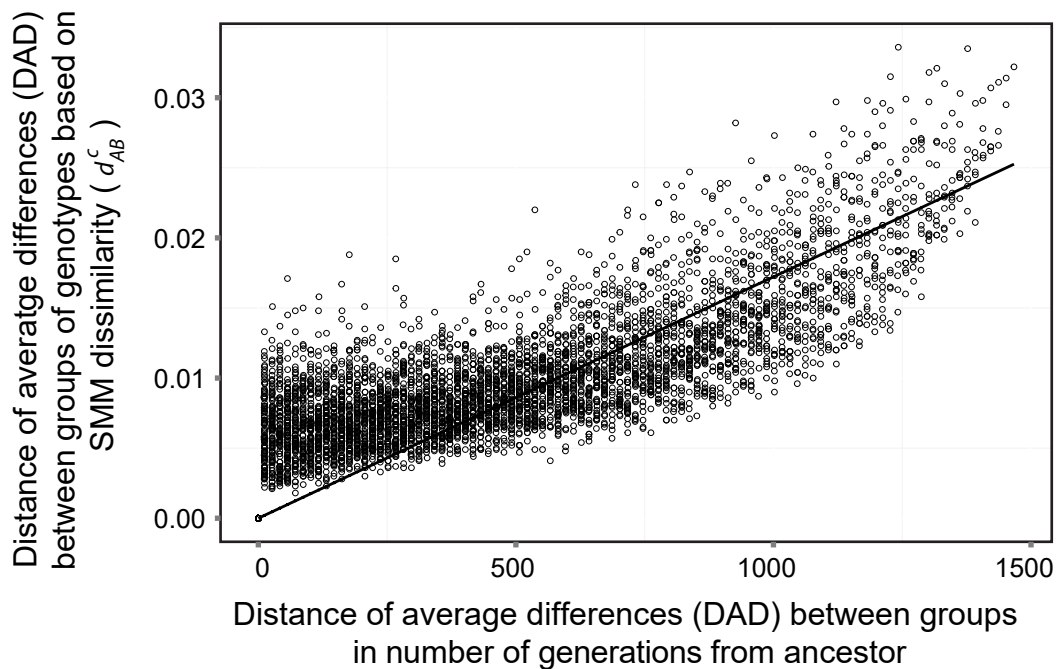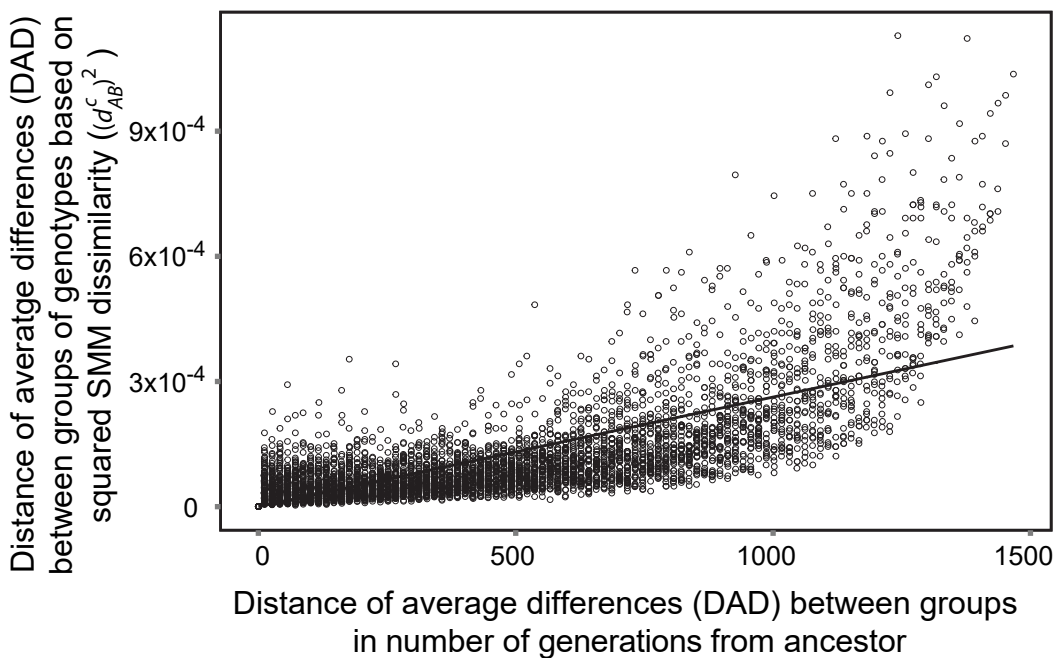

Figure 25. Kosman and Jokela

Supplement: Supplementary file 2 [file ECE3-9-4038-s002.pdf]
