# Supplementary material for: Dissimilarity of individual microsatellite profiles under different mutation models: Empirical approach
Source: Ecol Evol. 2019 Mar 19;9(7):4038–54. doi: 10.1002/ece3.5032 (PMC6467862; doi:10.1002/ece3.5032)
Supplement: Supplementary file 3 [file ECE3-9-4038-s003.docx]

**Dissimilarity of individual microsatellite profiles under different mutation models – empirical approach**

E Kosman and J Jokela

The following results demonstrate that a mode of SSR evolution (constant or variable mutation rate) has much stronger effect on implications regarding relationships of individuals than a way of measuring of an evolutionary difference between SSR alleles (absolute vs. squared difference).

**Table 1S**. Association between dissimilarity matrixes (below diagonal) and UPGMA dendrograms obtained with the corresponding dissimilarities (above diagonal) measured with Mantel tests for (**a**) *Cristatella mucedo* population; (**b**) collection of *Puccinia triticina* isolates; and (**c**) collection of *Blumeria graminis* isolates.

| **a** | SMMc2 | SMMv2 | SMMc | SMMv |
| --- | --- | --- | --- | --- |
| SMMc2 |  | 0.651 | 0.912 | 0.192 |
| SMMv2 | 0.662 |  | 0.841 | 0.725 |
| SMMc | 0.872 | 0.890 |  | 0.505 |
| SMMv | 0.457 | 0.936 | 0.814 |  |
|  |  |  |  |  |
| **b** | SMMc2 | SMMv2 | SMMc | SMMv |
| SMMc2 |  | 0.243 | 0.644 | 0.236 |
| SMMv2 | 0.476 |  | 0.623 | 0.808 |
| SMMc | 0.842 | 0.784 |  | 0.665 |
| SMMv | 0.442 | 0.979 | 0.805 |  |
|  |  |  |  |  |
| **c** | SMMc2 | SMMv2 | SMMc | SMMv |
| SMMc2 |  | 0.584 | 0.895 | 0.609 |
| SMMv2 | 0.675 |  | 0.608 | 0.939 |
| SMMc | 0.932 | 0.747 |  | 0.718 |
| SMMv | 0.621 | 0.944 | 0.765 |  |

SMMc = $d_{AB}^{c}$ dissimilarity for the stepwise mutation model with a constant rate of mutations (eq. 5);

SMMv = $d_{AB}^{v}$ dissimilarity for the stepwise mutation model with a variable rate of mutations (eq. 4);

SMMc2 = $d_{AB}^{c2}$ dissimilarity for the stepwise mutation model with a constant rate of mutations based on the squared differences between allele sizes;

SMMv2 = $d_{AB}^{v2}$ dissimilarity for the stepwise mutation model with a variable rate of mutations based on the squared differences between allele sizes.
