# Supplementary material for: Dissimilarity of individual microsatellite profiles under different mutation models: Empirical approach
Source: Ecol Evol. 2019 Mar 19;9(7):4038–54. doi: 10.1002/ece3.5032 (PMC6467862; doi:10.1002/ece3.5032)
Supplement: Supplementary file 4 [file ECE3-9-4038-s004.docx]

**Appendix**

***Generalization of Bruvo’s distance between SSR alleles***

Bruvo’s distance between SSR alleles (Bruvo *et al*., 2004) was suggested as a consequence of the generalized stepwise mutation model (SMM), in which mutations may result in an increase or decrease by any finite number of repeat units (Slatkin, 2002). Then a probability *m_k_* of mutation of size *k* (loss or addition of *k* repeat units) during one generation was determined using a symmetric geometric distribution with parameter *α* ($0<\alpha<1$):

$m_{k}=\frac{\mu}{2}\cdot\alpha\left( 1-\alpha\right)^{\left| k \right|-1}$., if $k\neq0$, and $1-\mu$, if $k=0$ ,

where *μ* is a constant mutation rate ($0<\mu<1$). Then $m_{-k}+m_{k}=\mu\cdot\alpha\left( 1-\alpha\right)^{\left| k \right|-1}$ , and

$$1-\mu\cdot\alpha\left( 1-\alpha\right)^{\left| k \right|-1} (A1)$$

is a probability of no mutations ($k=0$) or any mutation of size *l*, $l\neq k$ . Then Bruvo’s distance between two SSR alleles with differences *k* in the number of repeat units was determined from eq. 8 for $\mu=1$ and $\alpha=\frac{1}{2}$ (eq. 2 in Bruvo *et al*., 2004):

$$d_{a}=1-2^{-\left| k \right|} . (A2)$$

Such definition of distance between two alleles is a bit confusing. On the one hand, it is legitimate and reflects the premise that distance between alleles should not increase linearly as a function of difference between sizes of the corresponding alleles. However, there is nothing in common between probabilities in eq. 8 and distances in eq. 9. This is simply a case when two different phenomena can mathematically be expressed by the same equation. Below, we will further develop the idea of measuring dissimilarity between microsatellite alleles with non‑linear functions of the corresponding differences in allele sizes.

Let two alleles *A_s_* and *A_t_* at the same locus consist of *s* and *t* repeat units, respectively. We denote $=\left| s-t \right|$ , and $f(x)$, $x\geq0$, a function aimed at measuring dissimilarity $d_{st}^{f}$ between alleles *A_s_* and *A_t_* :

$$d_{st}^{f}=f\left( x \right)=f\left( \left| s-t \right| \right)=d^{f} (A_{s},A_{t}). (A3)$$

The following is a list of desirable properties of dissimilarity $d_{st}^{f}$ or function $f\left( x \right)$ assuming that a probability of transition of one SSR allele into another is described by the above model: (i) $0\leq f\left( x \right)\leq1$ ; (ii) $f\left( x \right)$ is a strictly increasing function; (iii) $f\left( 0 \right)=0$ and $\lim_{x\to\infty} f(x)=1$; and perhaps (iv) $f\left( x \right)$ is a concave function. The first three properties are easily interpreted because, in particular, it is natural to postulate that the lower the probability of evolution of one allele into another, the larger the dissimilarity between those alleles. The fourth property is generally optional, but we think desirable as it specifically means the following: for alleles *A_s_*, *A_t_* and *A_t+1_* distinction between dissimilarities $d_{st}^{f}$ and $d_{s,t+1}^{f}$ decreases with increasing difference $\left| s-t \right|$ in number of repeat units of *A_s_* and *A_t_*. More generally, if two alleles *A_t_* and *A_t+u_* with a fixed difference *u* in repeat units are more divergent from another given allele *A_s_* in terms of repeat units ($\left| s-t \right|$ increases), then distances between each of those alleles and the given allele ($d_{st}^{f}$ and $d_{s,t+u}^{f}$) become more similar.

Many functions meet all conditions (i) – (iv) sufficient for an adequate measure of dissimilarity between microsatellite alleles. Among them $f\left( x \right)=x/{max}$ or $d_{st}^{f}=\left| s-t \right|/{max}$ (*max* is a range of allele differences expressed by number of repeat units) that corresponds to our measure $\rho_{j}\left( A_{i},A_{k} \right)$ from eq. 2, and

$g\left( x \right)=1-\alpha^{-x}$ with $\alpha>1$ (A4)

that for $\alpha=2$ converts to the Bruvo’s distance $d_{a}$ from eq. 9. Another options can also be considered:

$h_{1}\left( x \right)=1-\frac{1}{\left( 1+x \right)^{\beta}}$ or $h_{2}\left( x \right)=1-\frac{\beta}{1+x}$ with $\beta>0$, (A5)

$r_{1}\left( x \right)=1-\frac{1}{1+\log_{\gamma} \left( 1+x \right)}$ or $r_{2}\left( x \right)=1-\frac{\gamma}{1+\ln(1+x)}$ with $\gamma>1$ (A6)

etc. The corresponding series of non-linear dissimilarity measures for microsatellite alleles within the same locus are obtained by substituting functions $g\left( x \right)$, $h_{1}\left( x \right)$, $h_{2}\left( x \right)$, $r_{1}\left( x \right)$ and $r_{2}\left( x \right)$ into eq. 10. These dissimilarities mainly differ in how fast and evenly they approach their maximum value 1. Thus, Bruvo’s estimate of dissimilarity between two SSR alleles (eq. 9) is only one among several other alternatives. The question whether selection of the most suitable approach in general and in each particular situation is possible needs further analysis, both theoretical and empirical, with simulated and real data. Alternatively, one could compare conceptually different approaches by analyzing the expected results obtained with each of them, and formulate the corresponding hypothesis to be tested with a particular empirical system. The latter seems more reasonable in study of natural populations with generally unknown mode of evolution.

Importantly, variable mutation rate at different SSR loci can be taken into account by choosing locus specific parameters *α*, *β* and *γ*. Finally, dissimilarity between two multilocus microsatellite genotypes can be calculated according to eq. 3, where differences between alleles $d_{st}^{f}$ (eq. 10) for a selected function *f* substitute $\rho_{j}\left( A_{i},A_{k} \right)$ from eq. 2 in the corresponding algorithm.
