# Supplementary material for: Dissimilarity of individual microsatellite profiles under different mutation models: Empirical approach
Source: Ecol Evol. 2019 Mar 19;9(7):4038–54. doi: 10.1002/ece3.5032 (PMC6467862; doi:10.1002/ece3.5032)
Supplement: Supplementary file 5 [file ECE3-9-4038-s005.pdf]

Figure 1S. Simulated haploid multilocus (8 loci) genotype data showing relationship of pairwise differences between genotypes in SMM dissimilarity and in distances to common ancestor sharing same alleles. Top panels shows the relationships for distances to ancestor measured as number of mutation events (relative scale from 0 to 1) and the bottom panel shows the same for distances to ancestor in number of generations (relative scale from 0 to 1). In both panels three dissimilarities are calculated based on Bruvo dissimilarity, absolute allele size difference and squared difference between alleles $(d_{AB}^{c})^2$ assuming constant mutation rate across loci.

Each simulation consisted of 100 lineages evolving between 10 and 900 generations (assigned from uniform random distribution). Each locus started with allele size of 200 and followed a random walk stepwise mutation process with a probability of 0.5 for mutation to take place. Mutation either increased or decreased allele size for one repeat with equal probability. Simulation recorded the number of mutation events and kept track of the allele size. All loci in the genotype were linked in the sense that they share the number of generations but independent in the sense that mutations were assigned randomly.

Lines show the fit of LS regression between the y and x variables and were used to calculate RMSE and R-squared. Regression was fitted without intercept.

Figure 2S. Distance of average differences (DAD) between groups of related haploid sexual genotypes shown against the pairwise differences (DAD distance) in number of generations between those groups of genotypes. Top panel shows DAD based on absolute SMM allele size differences (DAD with regard to d_{AB}^{c}) and bottom panel shows the DAD based on squared SMM allele size differences (DAD with regard to $(d_{AB}^{c})^2$).

Simulation generated 100 groups of 20 unlinked multilocus genotypes (8 loci). Each group consisted of loci that were similar in age. This was achieving by setting the selection criteria so that maximum age difference to ancestral allele among the alleles was not more than 9 generations. Groups were set to differ by age. Groups did not evolve, they were simulated de novo each time. In this sense they represent unconnected populations that have a different evolutionary history. The average difference in age between groups was set to 15 generations. The “youngest” allele was 20 generations from ancestor and the oldest allele was 1514 generations from the ancestor. Ancestral allele size was 200 repeats. Mutation rates and the mutation model as in Figure 1S.
